# ICTV Virus Taxonomy Profile: *Cystoviridae*

**DOI:** 10.1099/jgv.0.000928

**Published:** 2017-09-20

**Authors:** Minna M. Poranen, Sari Mäntynen, ICTV Report Consortium

**Affiliations:** ^1^​ Department of Biosciences, University of Helsinki, 00014 Helsinki, Finland; ^2^​ Centre of Excellence in Biological Interactions, Department of Biological and Environmental Science and Nanoscience Center, University of Jyväskylä, 40014 Jyväskylä, Finland

**Keywords:** *Cystoviridae*, ICTV, taxonomy, Pseudomonas phage phi6

## Abstract

The family *Cystoviridae* includes enveloped viruses with a tri-segmented dsRNA genome and a double-layered protein capsid. The innermost protein shell is a polymerase complex responsible for genome packaging, replication and transcription. Cystoviruses infect Gram-negative bacteria, primarily plant-pathogenic *Pseudomonas syringae* strains. This is a summary of the International Committee on Taxonomy of Viruses (ICTV) Report on the taxonomy of the *Cystoviridae,* which is available at http://www.ictv.global/report/cystoviridae.

## Abbreviation

PC, polymerase complex.

## Virion

**Fig. 1.**
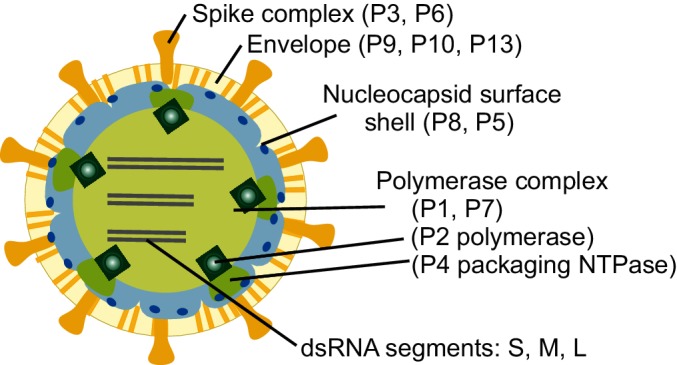
Schematic presentation of cystovirus particle (Pseudomonas phage phi6) with location of virion proteins.

The spherical virion of a cystovirus has three structural layers (Fig. 1 and Table 1). The outermost layer is the lipid bilayer envelope, consisting of host-derived phospholipids [1] and four virally encoded integral membrane proteins (P6, P9, P10, P13). Host attachment spikes (formed by P3) are anchored to the envelope via fusogenic protein P6 (2). The envelope encloses the nucleocapsid, consisting of two concentric protein layers: the nucleocapsid surface shell and the polymerase complex (PC) core [2]. The nucleocapsid surface shell contains 200 copies of protein P8 trimers arranged into a T=13 icosahedral lattice [3]. The internal PC core consists of four protein species: the major structural protein P1, the RNA-dependent RNA polymerase P2 [4], the hexameric packaging NTPase P4 [5] and the assembly cofactor P7 [6]. The structural framework of the PC core is formed by 120 copies of protein P1, arranged as asymmetric dimers on a T=1 icosahedral lattice.

**Table 1.** Characteristics of the family *Cystoviridae*

Typical member:	Pseudomonas phage phi6 (Segment S, M12921; Segment M, M17462; Segment L, M17461), species *Pseudomonas virus phi6*, genus *Cystovirus*
Virion	Enveloped virions (~85 nm) with two concentric, icosahedrally symmetric protein layers: the nucleocapsid surface shell (T=13) and the polymerase complex core (T=1). Spikes protrude from the virion surface
Genome	Three segments of linear, double-stranded RNA, totaling 13.4 kbp, encoding 13 genes
Replication	Single-stranded genomic precursor molecules are packaged into the viral polymerase complex. The packaged RNA molecules are replicated and transcribed within the particle
Translation	Viral proteins are translated from polycistronic messenger RNA molecules
Host range	Gram-negative bacteria, mostly *Pseudomonas* species
Taxonomy	One genus (*Cystovirus*) and one species (*Pseudomonas virus phi6*)

## Genome

**Fig. 2.**
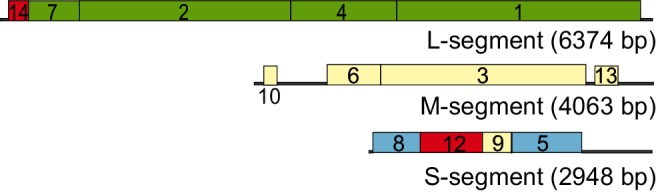
Genome organization of Pseudomonas phage phi6. The gene and protein numbers are the same. Colourings indicate genes encoding constituents of the polymerase complex (green), nucleocapsid (blue), envelope-associated proteins (cream) and non-structural proteins (red).

Cystoviruses have three segments of linear, double-stranded RNA, named according to their size as L (large, 6.4 kbp), M (medium, 4.1 kbp) and S (small, 2.9 kbp) (Fig. 2). One copy of each genome segment is encapsidated in a virion. In each segment, genes are clustered into functional groups. The coding regions are flanked by terminal non-coding regions containing signals for genome packaging and replication [2].

## Replication

Infection is initiated when the virion adsorbs to host pili [7]. As the pilus retracts, the virus particle reaches the bacterial outer membrane. Subsequently, envelope protein P6 induces fusion between the viral envelope and the host outer membrane, resulting in the release of the nucleocapsid into the periplasmic space [8]. The peptidoglycan layer is digested by virion-associated lytic enzyme P5 and the nucleocapsid is exposed to the host cytoplasmic membrane. Via an endocytic-like process, the nucleocapsid enters the cytoplasm [9]. The virion-associated RNA polymerase [4] is activated and viral transcription commences. Transcription is semi-conservative [10] and produces full-length, polycistronic copies of the genome segments (Table 1). Early in the infection approximately equal amounts of messenger RNA molecules are produced from each genome segment. The early proteins translated from the L transcript assemble to form empty PC cores [6]. One copy of each type of transcript is packaged into an empty PC core, ultimately triggering the negative-strand synthesis within the core [6]. After replication, a second round of transcription initiates, resulting in the predominant production of S and M transcripts that direct the production of late proteins needed in virion assembly [2]. The nucleocapsid surface shell assembles on the genome-containing polymerase complex [6]. Finally, the envelope derived from the host plasma membrane [1] encloses the nucleocapsid and spikes attach on the virion surface. Ultimately, mature virions are released upon virus-induced host cell lysis [7].

## Taxonomy

A single genus, *Cystovirus*, with one species, *Pseudomonas virus phi6*.

## Resources

Full ICTV Online (10th) Report: http://www.ictv.global/report/cystoviridae.
